# The Book World of Medicine and Science

**Published:** 1897-05-08

**Authors:** 


### THE BOOK WORLD OF MEDICINE AND SCIENCE.

DIPHTHERIA AND ANTI-TOXIN. By NESTOR TIRARD, M.D., Physician to King's College Hospital and Evelina Hospital for Children. (Longmans. 1897.)

To those with no special knowledge of the modern treatment of the disease, and no time for bibliographical research, this volume will prove useful, not merely for a single perusal, but for ready reference for some years. It does not profess, evidently, to be an exhaustive monograph, but may therefore prove of more practical utility for general use. We should have been glad to see a little more information on the bacteriology, and more stress laid on the nature of the toxin of diphtheria; a sketch of the principles on which anti-toxins are prepared, and some remarks on the various qualities of anti-toxin offered for use would have been of value; but Dr. Tirard has preferred, perhaps wisely, to base his deductions largely on the cases treated at the Evelina under his own observation, these cases having been treated with Behring's anti-toxin. As to the great value of anti-toxin, the author has no hesitation; his summary on this head is judicial and reserved; he might even have emphasised more fully such points as the marked rapidity with which membrane separates under its use, and the rapid improvement from an apparent collapse which many severe cases show. His cases do not appear to have exhibited the amount of reaction (joint pain, temperature, and rashes) which some observers have recorded, perhaps with the employment of other forms of anti-toxin; but it has yet to be proved that such reaction is of any importance. Great stress has been laid by Mr. Lennox Browne on the occurrence of albuminuria as a result of anti-toxin, but Dr. Tirard adds the weight of his own experience to the already large array of counter evidence. He also points out with a judicious fulness that too much importance is attached to albuminuria when only slight; it does not give rise to any distinct symptoms, and does not leave any permanent trouble, as in scarlet fever. He would, on the other hand, have done well (following Goodall) to impress on his readers the importance of anuria as a symptom of bad augury.

On the subject of diagnosis it is excellent advice that we should in all cases of suspected diphtheria test the knee-jerks carefully; it is never necessary to remove the patient from bed for this purpose. Dr. Tirard shows a strong leaning towards intubation, and devotes a proper proportion of space to describing its performance and after treatment, but he scarcely rates sufficiently the danger of a tube being coughed up, when, as in private practice, there is no house surgeon at hand to replace it. It is probable, as he points out, that the use of anti-toxin will be to increase that of intubation as compared with tracheotomy.

The style of the book is simple, occasionally too simple, and concise. A short bibliography would increase its value. As far as it goes we can sincerely recommend the volume for the shelf of the general practitioner.

QUANTITATIVE ESTIMATION OF URINE: NEW SYSTEM OF RAPID ANALYSIS. By J. BARKER SMITH, L.R.C.P. London. (Ballière, Tindall, and Cox, 21, King William Street, Strand, W.C. 1897. Price 1s. 37 pages.)

This paper describes the author's ingenious method for the quantitative analysis of urine. The first group of tests includes those for estimating acidity, urea, sugar, total urates, phosphates, ammonia, &c. The second group includes the tests for albumen, biliary salts, peptones, &c. The method is very rapid. The whole procedure occupies less than an hour, and does not need a special apparatus. The method should prove valuable to medical men, and this pamphlet, in which the steps are carefully explained, is well worth studying.

